# Rhabdomyolysis, lactic acidosis, and multiple organ failure during telbivudine treatment for hepatitis B: a case report and review of the literature

**DOI:** 10.1186/s13256-017-1498-6

**Published:** 2017-11-27

**Authors:** Jinxin Zheng, Minggui Deng, Xiaoliang Qiu, Zhong Chen, Duoyun Li, Xiangbin Deng, Qiwen Deng, Zhijian Yu

**Affiliations:** 1 Department of Infectious Diseases and Shenzhen Key Lab for Endogenous Infection, Shenzhen Nanshan Hospital, Shenzhen University, No 89, Taoyuan Road, Nanshan District, Shenzhen, 518052 China; 2 Department of Medicine, Marshall University School of Medicine, 1600 Medical Center Drive, Huntington, WV 25701 USA

**Keywords:** Hepatitis B, Lactic acidosis, Multiple organ failure, Rhabdomyolysis, Telbivudine

## Abstract

**Background:**

Telbivudine can cause severe side effects, including myositis, neuritis, rhabdomyolysis, and lactic acidosis. However, reported cases of telbivudine leading to multiple organ failure are rare. Here, we report a case of telbivudine-induced severe polymyositis, lactic acidosis, and multiple organ failure.

**Case presentation:**

A 30-year-old Chinese man with hepatitis B virus infection received antiviral treatment with 600 mg of telbivudine daily for more than 11 months. He developed progressive weakness and myalgia, and subsequently experienced palpitations, chest tightness, lethargy, hypotension, and hypoxemia. Blood tests showed markedly elevated levels of alanine aminotransferase (955 U/L), aspartate aminotransferase (1375 U/L), blood urea nitrogen (14.9 mmol/L), creatine kinase (peak at 8050 U/L), and blood lactate (>20.0 mmol/L). His symptoms improved after continuous renal replacement therapy and short-term methylprednisolone treatment. Hyperbaric oxygen therapy, physical therapy, and rehabilitation for more than 2 months led to recovery of muscle strength to the normal range.

**Conclusions:**

We conclude that continuous renal replacement and steroid therapies play key roles in stabilizing telbivudine-induced severe rhabdomyolysis, lactic acidosis, and multiple organ failure. Hyperbaric oxygen, physical therapy, and rehabilitation may aid in functional recovery after the acute phase of lactic acidosis and organ failure.

## Background

One of the most common infectious diseases in the world, chronic hepatitis B is a serious public health problem in China [1]. Pathogenesis of hepatitis B virus (HBV) is still not completely clear. Currently, the standard therapy for chronic hepatitis B is antiviral treatment with nucleoside analogs, including lamivudine, adefovir, entecavir, telbivudine, or tenofovir.

In October 2006, the L-nucleoside analog telbivudine at a dose of 600 mg/day was approved for HBV treatment. Compared to lamivudine, telbivudine showed a lower rate of drug resistance and higher rate of hepatitis B envelope antigen (HBeAg) seroconversion [2]. However, a clinical trial revealed myopathy to be an adverse effect [2]. Telbivudine therapy led to grade 3/4 creatine kinase (CK) elevation in 0.3 to 5% of patients [3], as well as accelerated muscle toxicity in patients with pre-existing muscle damage [4]. Telbivudine can also cause fatal rhabdomyolysis and lactic acidosis [5, 6].

Telbivudine-associated elevation of CK levels, myopathy, hyperlactatemia, and rhabdomyolysis have been frequently reported, whereas reports of multiple organ failure (MOF) are rare. Here, we describe a patient who was treated for 11 months with telbivudine for HBV and subsequently developed rhabdomyolysis, lactic acidosis, and MOF.

## Case presentation

A 30-year-old Chinese man who had been HBeAg-positive for at least 10 years received telbivudine (Novartis Pharma AG, Basel, Switzerland) at a dosage of 600 mg once daily for 1 year before he presented to our hospital. He had no previous history of antiviral therapy. After 11 months of treatment, he complained of progressive pain and weakness in his lower extremities, poor appetite, nausea, and vomiting. Biochemical tests showed that his serum CK level had increased to 1000 U/L. Telbivudine was stopped immediately.

He was admitted to a ward with worsening symptoms. Laboratory tests were positive for hepatitis B surface antigen and HBeAg, but HBV deoxyribonucleic acid (DNA) was not detected. His serum CK level was elevated to 1220 U/L. Electromyography indicated slow nerve conduction velocity in both lower extremities. One week after admission, he developed shortness of breath, dyspnea, chest pain, malaise, and cold limbs with terminal cyanosis. The muscle strengths of his upper and lower extremities on the Medical Research Council scale were 2/5 and 1/5, respectively.

He was transferred to our intensive care unit, where he was found to have hypotension with a blood pressure of 82/43 mmHg. Oxygen saturation decreased to 80% despite nasal oxygen inhalation at a flow rate of 5 L/minute. Biochemical tests showed increased levels of alanine aminotransferase (ALT, 955 U/L), aspartate aminotransferase (AST, 1375 U/L), blood urea nitrogen (BUN, 14.9 mmol/L), serum CK (peak at 8050 U/L), and lactate dehydrogenase (LDH, 2040 U/L; Table 1). He was put on continuous mechanical ventilation. Arterial blood gas analysis revealed lactic acidosis, with a partial pressure of oxygen (PO_2_) of 63 mmHg, partial pressure of carbon dioxide in arterial blood (PaCO_2_) of 24.7 mmHg, pH of 7.275, bicarbonate (HCO_3_^-^) of 11.6 mmol/L, and blood lactate level exceeding 20.0 mmol/L (upper limit of normal, 2.2 mmol/L).

**Table 1:** Major laboratory results according to days since hospital admission

Parameter	1 d	8 d	12 d	14 d	16 d	18 d	24 d	28 d	38 d	49 d	55 d	62 d	74 d	84 d	94 d	105 d
TB (umol/L)	13.9	15.1	19.1	22.5	–	14.9	–	14.8	12.3	9.9	12.5	12.0	6.7	6.6	8.6	10.9
ALT (U/L)	25.0	80.0	350.0	955.0	–	166.0	–	73.0	48.0	32.0	34.0	25.0	11.0	15.0	14.0	17.0
AST (U/L)	74.0	106.0	216.0	1375.0	217.0	136.0	29.0	25.0	27.0	28.0	33.0	25.0	22.0	20.0	19.0	21.0
ALB (g/L)	38.0	32.3	35.4	33.7	–	31.8	–	38.8	41.0	37.8	38.2	38.7	37.6	38.5	38.9	41.0
LDH (U/L)	439.0	438.0	494.0	2040.0	724.0	693.0	548.0	368.0	239.0	–	169.0	–	–	–	–	–
CK (U/L)	1220.0	1148.0	416.0	3568.0	8050.0	2440.0	124.0	73.0	56.0	54.0	84.0	136.0	94.0	189.0	95.0	187.0
CK-MB (U/L)	17.0	22.0	13.6	36.9	43.3	26.3	13.4	12.3	9.6	9.2	10.6	10.7	10.0	10.0	10.1	12.1
Lac (mmol/L)	2.2	20.0	5.1	2.1	2.3	2.2	1.7	1.5	1.4	–	1.4	–	1.7	–	2.2	1.2
WBC (× 10^9^/L)	6.7	21.1	14.0	13.4	10.5	12.7	12.2	11.3	10.0	7.2	–	7.6	6.4	5.5	6.4	5.8
RBC (× 10^12^/L)	4.2	4.2	3.1	2.9	2.8	3.0	3.5	3.7	4.1	4.0	–	4.1	4.0	4.1	4.3	4.3
PLT (× 10^9^/L)	210.0	329.0	48.0	48.0	46.0	82.0	185.0	182.0	217.0	187.0		246.0	236.0	222.0	197.0	209.0
BUN (mmol/L)	3.6	14.9	11.6	10.5	9.0	5.5	4.3	5.1	4.6	2.1	1.9	1.2	2.5	2.4	2.0	2.0
PT (seconds)	–	16.7	16.5	16.9	17.3	14.7	12.9	–	–	–	–	–	–	–	–	–
UMS (level)	4.0	2.0	3.0	3.0	3.0	3.0	2.0	3.0	3.0	3.0	4.0	4.0	4.0	5.0	5.0	5.0
LMS (level)	3.0	1.0	1.0	1.0	1.0	1.0	1.0	2.0	3.0	3.0	3.0	3.0	3.0	4.0	4.0	4.0

Normal ranges are given in parentheses as follows: *ALB* albumin (35–55 g/L), *ALT* alanine aminotransferase (5–45 U/L), *AST* aspartate aminotransferase (5–45 U/L), *BUN* blood urea nitrogen (1.7–7.1 mmol/L), *CK* creatine kinase (10–195 U/L), *d* days, *LDH* lactate dehydrogenase (80–285 U/L), *LMS* muscle strength of lower extremities (according to the Medical Research Council scale), - not detected, *CK-MB* cardiac creatinine phosphokinase isoenzyme (0–24 U/L), *Lac* lactate (0–2.2 mmol/L), *PLT* platelet (100–300 × 10^9^/L), *PT* prothrombin time (10–14.5 seconds), *RBC* red blood cell (4.0–5.5 × 10^12^/L), *TB* total bilirubin (1.7–22.5 μmol/L), *UMS* muscle strength of upper extremities (according to the Medical Research Council scale), *WBC* white blood cell (4.0–10.0 × 10^9^/L)

**Fig. 1:**
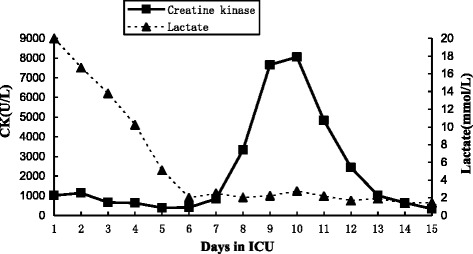
Patient’s serum lactate and creatine kinase levels during his stay in our intensive care unit

**Fig. 2:**
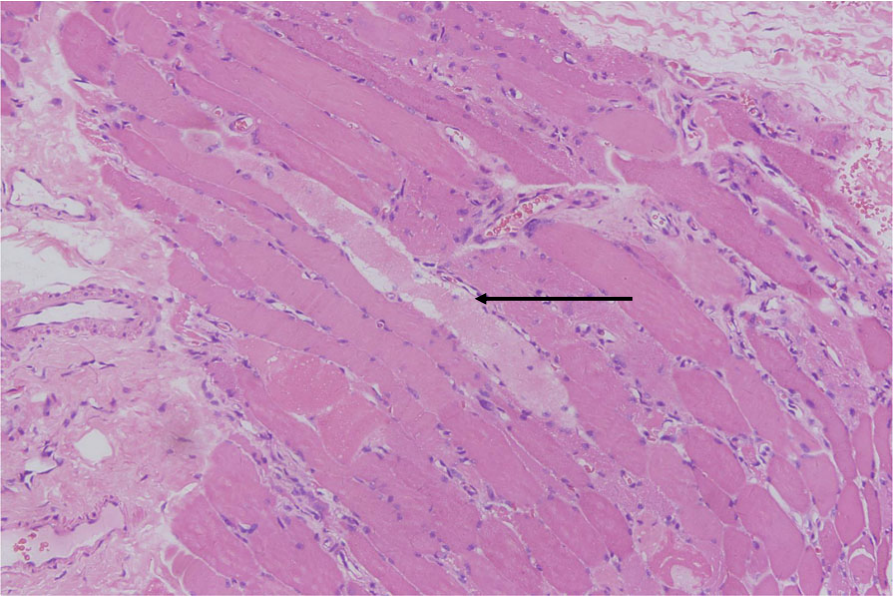
Muscle biopsy from left gastrocnemius muscle. Hematoxylin and eosin-stained paraffin section of muscle biopsy. Multiple degenerating atrophic myofibers (*arrow*) and numerous necrotic myofibers infiltrated by macrophages were observed, indicating multiple nerve root inflammation and polymyositis (original magnification, ×200)

Continuous renal replacement therapy (CRRT) was maintained for 10 days, and methylprednisolone was initiated at 80 mg twice daily. These treatments led to improvements in his chest pain, dyspnea, and laboratory parameters (Table 1 and Fig. 1), but did not resolve his poor muscle strength. A biopsy of his left gastrocnemius muscle showed multiple nerve root inflammation and polymyositis with multiple degenerating atrophic myofibers and numerous necrotic myofibers infiltrated with macrophages (Fig. 2).

**Fig. 3:**
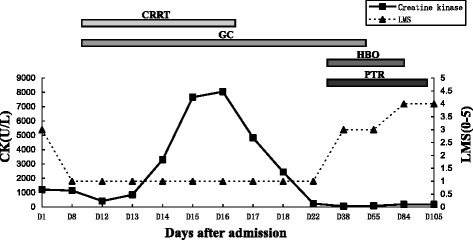
Relationship between primary treatment, creatine kinase level, and muscle strength. *CK* creatine kinase, *CRRT* continuous renal replacement therapy (for 8 days), *D 1* Day 1, *GC* glucocorticoid therapy (including methylprednisolone, dexamethasone, and prednisone, for 44 days), *HBO* hyperbaric oxygen therapy (for 40 days), *LMS* muscle strength of lower extremities (according to the Medical Research Council scale), *PTR* physical therapy and rehabilitation (for 70 days)

He was transferred from our intensive care unit to our infectious disease ward. His HBV DNA was elevated at 8.48 × 10^4^ IU/ml. Antiviral treatment with adefovir dipivoxil (10 mg once daily) was started. To improve muscle strength, he received simultaneous hyperbaric oxygen (HBO) therapy for approximately 40 days, as well as physical therapy and rehabilitation (PTR; including neuromuscular electrical stimulation therapy, comprehensive paraplegic limb training, electro-acupuncture treatment, and infrared therapy) for approximately 70 days. His muscle strength gradually improved, and his serum ALT, AST, CK, and LDH levels returned to normal. He was then discharged. Six months after discharge, his muscle strength was within the normal range. He resumed his work as a taxi driver 1 year after discharge. Relationships between primary treatment, CK levels, and muscle strength are shown in Fig. 3.

## Discussion

A new nucleoside analog for treatment of chronic hepatitis B, telbivudine inhibits synthesis of second-strand DNA through competing with the natural substrate of DNA polymerase (reverse transcriptase) [7, 8]. In one phase II clinical trial, telbivudine monotherapy reduced serum HBV DNA and ALT levels more rapidly than lamivudine monotherapy [9]. Telbivudine is currently regarded as an efficacious, well-tolerated, and safe drug for HBV treatment [10]. Despite these advantages, there have been frequent reports of adverse effects, including elevation of CK levels, myopathy, and neuropathy, associated with telbivudine treatment, particularly when combined with pegylated interferon [11]. The GLOBE study showed that telbivudine was more frequently associated with asymptomatic hyper-CK-emia compared to lamivudine (12.9 versus 4.1%) [2]. Among patients treated with telbivudine, 12 to 14% reported fatigue and malaise, 9% reported asymptomatic hyper-CK-emia, and 0.5% reported myopathy during treatment [12].

Male sex, younger age, and HBeAg negativity were independent predictors of elevated CK levels in patients receiving telbivudine treatment in China [3]. Recently, Chen *et al*. found that male sex and lower baseline estimated glomerular filtration rate were significant risk factors for elevation of CK levels during telbivudine treatment [13]. In our patient, the CK level was markedly elevated (peak at 8050 U/L). Male sex may have been associated with prolonged exercise or acceleration of pre-existing muscle damage, a phenomenon that was reported previously [4].

Several studies reported telbivudine-related myopathy and neuropathy, but these side effects were typically mild and reversible [14–17]. To date, reports of telbivudine-induced severe side effects, such as rhabdomyolysis, hyperlactatemia, lactic acidosis, or MOF, have been very rare. Jin *et al*. described a 36-year-old man who developed severe refractory lactic acidosis during telbivudine monotherapy. He fully recovered after 16 weeks of hemodialysis and glucocorticosteroid treatment [6]. Wang *et al*. described a patient who developed hyperlactatemia during telbivudine treatment and was successfully treated with continuous venovenous hemodiafiltration [18]. However, another patient with telbivudine-induced rhabdomyolysis developed acute renal failure and metabolic acidosis by day 18 after admission and, despite hemofiltration, died within 24 hours of developing these symptoms [5]. Table 2 lists characteristics of previously reported patients who developed severe adverse effects during telbivudine treatment. As these conditions are potentially fatal, immediate hemodialysis and glucocorticosteroid therapy are indicated.

**Table 2:** Literature review of patients who developed severe adverse events during telbivudine treatment

Patient number	Age (years)	Adverse effect	Liver condition	Tx time (months)	Peak CK (U/L)	Peak lactate (mmol/L)	Prognosis	Reference
1	27	Myopathy	CHB	3	3243	–	Resolved	[4]
2	67	Myopathy	CHB	20	4775	–	Resolved	[14]
3	25	Myopathy	CHB	6	1614	–	Resolved	[15]
4	28	Myopathy	CHB	9	788	–	Resolved	[16]
5	25	Myopathy	CHB	13	2992	–	Resolved	[16]
6	68	Myopathy	CHB	2	237	–	Resolved	[17]
7	35	Rhabdomyolysis, lactic acidosis	CHB	11	3683	> 12.0	Resolved	[6]
8	26	lactic acidosis	CHB	12	4151	11.3	Resolved	[18]
9	30	Rhabdomyolysis, lactic acidosis, organ failure	CHB	11	8050	> 20.0	Resolved	This paper
10	48	Rhabdomyolysis, metabolic acidosis, organ failure	Cirrhosis	9	3246	–	Death	[5]

*CHB* chronic hepatitis B, *CK* creatine kinase, *Tx* treatment, lactate mmol/L × 9.608 = mg/dL

The risk factors for telbivudine-induced rhabdomyolysis, hyperlactatemia, lactic acidosis, or MOF are still unclear. A previous study found telbivudine appears to cause accelerated muscle toxicity if given to patients who already have muscle damage [4]. Another research found that in liver transplant recipients, telbivudine-induced polyneuropathy/myopathy maybe due to diabetes [19]. When it comes to lactic acidosis or hyperlactatemia caused by telbivudine, Jin *et al*. found that a case with telbivudine-induced lactic acidosis had a history of hypokalemic periodic paralysis, but the relationship between the pre-existence of myopathy and telbivudine treatment was uncertain [6]. However, like the case of another previous study, our patient also had no heavy exercise or heavy drinking, and had no history of diabetes mellitus [18]. Thus the risk factors for telbivudine-induced lactic acidosis or MOF are still ambiguous and need to be further explored. The case in this study developed severe telbivudine-induced rhabdomyolysis, lactic acidosis, and MOF; the reason maybe that his elevated CK levels and rhabdomyolysis were not discovered in a timely manner because he received no regular reexamination. Although telbivudine was stopped, the rhabdomyolysis continue progressed and developed to lactic acidosis and MOF. So during the telbivudine treatment for HBV, except for detecting the possible risk factors associated with telbivudine-induced rhabdomyolysis or lactic acidosis, monitoring for muscular abnormalities, CK levels, and rhabdomyolysis is also very important for these cases.

Mechanisms underlying telbivudine-induced myopathy and lactic acidosis remain unclear. Whereas some studies showed that telbivudine may cause mitochondrial toxicity and dysfunction, which might lead to myopathy and lactic acidosis [20], *in vitro* studies indicated no effect of the drug on lactic acid production, mitochondrial DNA content, or morphology [21]. Recently, Hernández-Laín *et al*. identified a novel *RRM2B* gene variant associated with telbivudine-induced mitochondrial myopathy [22]. Therefore, the mechanism remains to be elucidated.

In this report, we describe the case of 30-year-old man with telbivudine-induced severe side effects, including rhabdomyolysis, lactic acidosis, and MOF. His condition deteriorated rapidly after admission, with shortness of breath, muscle weakness, hypotension, hypoxia, and elevated CK levels within 1 week. Several reasons may account for the severe effects. Physicians may not have paid enough attention to the elevation of CK levels and myopathy during gastroenterology follow up. Underlying causes of his worsening condition were not immediately confirmed. CRRT and immunosuppressive therapy may not have been started as early as possible. Despite delays in diagnosis and treatment, our patient improved after CRRT and methylprednisolone therapy, which played key roles in the telbivudine-induced rhabdomyolysis, lactic acidosis, and MOF.

The role of glucocorticoid in the treatment of telbivudine-induced rhabdomyolysis, lactic acidosis, and MOF are unclear. In this study, we found our patient’s elevated lactate level in his blood and rhabdomyolysis improved significantly after CRRT and methylprednisolone therapy. But in another study, a patient with telbivudine-induced rhabdomyolysis only received hemofiltration therapy, the patient did not receive glucocorticoid therapy, and died within 24 hours [5]. Thus, the CRRT and glucocorticoid therapy may play key roles in stabilizing telbivudine-induced severe rhabdomyolysis, lactic acidosis, and MOF. Another study found that low-dose glucocorticoid for a short period of time may help to return the blood lactate level to normal, and the current study also indicated that CRRT combined with methylprednisolone therapy help to reduce the blood lactate level to normal.

However, we found that low-dose glucocorticoid therapy could not improve the poor muscle strength of our patient [6]. HBO therapy, which is very helpful in optic neuritis [23], can be used to treat myositis [24], although there have been no reports of HBO therapy for telbivudine-induced myositis or neuritis. A biopsy confirmed multiple nerve root inflammation and polymyositis in our patient, who subsequently received HBO therapy for more than 1 month and PTR for more than 2 months. Our patient gradually recovered, indicating that these interventions may be helpful in the treatment of telbivudine-induced myositis and neuritis. To the best of our knowledge, the patient in this study was the first case which experienced myopathy, rhabdomyolysis, lactic acidosis, shock, and MOF during telbivudine treatment and be treated successfully.

## Conclusions

Telbivudine can cause severe side effects, including myositis, neuritis, rhabdomyolysis, lactic acidosis, and even MOF. Risk of severe side effects is especially pronounced in younger male or highly active patients. CRRT and glucocorticoid therapy should be given as soon as possible after diagnosis, while HBO and PTR may be helpful for myositis and neuritis. Patients should be closely monitored for CK levels, myopathic symptoms, and blood lactate levels during telbivudine treatment.

## Abbreviations

ALT: Alanine aminotransferase
AST: Aspartate aminotransferase
BUN: Blood urea nitrogen
CK: Creatine kinase
CRRT: Continuous renal replacement therapy
HBeAg: Hepatitis B envelope antigen
HBO: Hyperbaric oxygen
HBV: Hepatitis B virus
HCO_3_^-^: Bicarbonate
LDH: Lactate dehydrogenase
MOF: Multiple organ failure
PaCO_2_: Partial pressure of carbon dioxide in arterial blood
PO_2_: Partial pressure of oxygen
PTR: Physical therapy and rehabilitation

## References

[1] Sun J, Hou JL. Management of chronic hepatitis B: experience from China. J Viral Hepat. 2010;17:10–7. 10.1111/j.1365-2893.2010.01274.x 20586929

[2] Liaw YF, Gane E, Leung N, *et al.* 2-Year GLOBE trial results: telbivudine is superior to lamivudine in patients with chronic hepatitis B. Gastroenterology. 2009;136:486–95. 10.1053/j.gastro.2008.10.026 19027013

[3] Zou XJ, Jiang XQ, Tian DY. Clinical features and risk factors of creatine kinase elevations and myopathy associated with telbivudine. J Viral Hepat. 2011;18:892–6. 10.1111/j.1365-2893.2010.01412.x 22093034

[4] Finsterer J, Ay L. Myotoxicity of telbivudine in pre-existing muscle damage. Virol J. 2010;7:323–5. 10.1186/1743-422X-7-323 PMC2998482 21083916

[5] Dang SS, Gao N, Zhang X, Jia XL. Rhabdomyolysis in a 48-Year-Old Man With Hepatitis B-Induced Cirrhosis. Am J Med Sci. 2011;342:73–5. 10.1097/MAJ.0b013e31821a520e 21642814

[6] Jin JL, Hu P, Lu JH, *et al.* Lactic acidosis during telbivudine treatment for HBV: A case report and literature review. World J Gastroenterol. 2013;19:5575–80. 10.3748/wjg.v19.i33.5575 PMC3761113 24023503

[7] Nash K. Telbivudine in the treatment of chronic hepatitis B. Adv Ther. 2009;26:155–69. 10.1007/s12325-009-0004-y 19225726

[8] Hernandez-Santiago B, Placidi L, Cretton-Scott E, *et al.* Pharmacology of beta-L-thymidine and beta-L-2′-deoxycytidine in HepG2 cells and primary human hepatocytes: relevance to chemotherapeutic efficacy against hepatitis B virus. Antimicrob Agents Chemother. 2002;46:1728–33. 10.1128/AAC.46.6.1728-1733.2002 PMC127241 12019082

[9] Lai CL, Gane E, Liaw YF, *et al.* Telbivudine versus lamivudine in patients with chronic hepatitis B. N Engl J Med. 2007;357:2576–88. 10.1056/NEJMoa066422 18094378

[10] Amarapurkar DN. Telbivudine: a new treatment for chronic hepatitis B. World J Gastroenterol. 2007;13:6150–5. 10.3748/wjg.v13.i46.6150 PMC4171223 18069753

[11] Fleischer RD, Lok AS. Myopathy and neuropathy associated with nucleos(t)ide analog therapy for hepatitis B. J Hepatol. 2009;51:787–91. 10.1016/j.jhep.2009.06.011 19665816

[12] Matthews SJ. Telbivudine for the management of chronic hepatitis B virus infection. Clin Ther. 2007;29:2635–53. 10.1016/j.clinthera.2007.12.032 18201580

[13] Chen L, Cheng C, Chen BC, Zhao Y, Zhang JM, Wang B. Cumulative incidence and risk factors of creatine kinase elevation associated with telbivudine. Eur J Clin Pharmacol. 2016;72:235–41. 10.1007/s00228-015-1978-9 26566799

[14] Caroleo B, Galasso O, Staltari O, *et al.* Muscular damage during telbivudine treatment in a chronic hepatitis B patient. Muscles Ligaments Tendons J. 2011;1:57–60. PMC3666470 23738248

[15] Wang M, Da YW, Cai HD, Lu Y, Wu LY, Jia JP. Telbivudine myopathy in a patient with chronic hepatitis B. Int J Clin Pharm. 2012;34:422–5. 10.1007/s11096-012-9633-3 22527478

[16] Kim EH, Park H, Lee KH, Ahn SH, Kim SM, Han KH. Two cases of telbivudine-induced myopathy in siblings with chronic hepatitis B. Clin Mol Hepatol. 2013;19:82–6. 10.3350/cmh.2013.19.1.82 PMC3622860 23593614

[17] Lee SW, Jang JH, Kim BJ. Dysphagia could be the first presenting symptom of telbivudine-induced myopathy. Intern Med J. 2013;43:1048–9. 10.1111/imj.12237 24004397

[18] Wang YH, Wu BQ, Liu H. Continuous venovenous hemodiafiltration for hyperlactatemia caused by telbivudine in a patient with chronic hepatitis B: A case report and update review. J Dig Dis. 2015;16:164–7. 10.1111/1751-2980.12173 25043654

[19] Turan I, Yapali S, Bademkiran S, *et al.* Telbivudine in Liver Transplant Recipients: Renal Protection Does Not Overcome the Risk of Polyneuropathy and Myopathy. Liver Transpl. 2015;21:1066–75. 10.1002/lt.24131 25845464

[20] Fontana RJ. Side effects of long-term oral antiviral therapy for hepatitis B. Hepatology. 2009;49:S185–95. 10.1002/hep.22885 19399802

[21] Standring DN, Bridges EG, Placidi L, *et al.* Antiviral beta-L-nucleosides specific for hepatitis B virus infection. Antivir Chem Chemother. 2001;12:119–29. 11594678

[22] Hernández-Laín A, Guerrero AM, Domínguez-González C, *et al.* A novel *RRM2B* gene variant associated with Telbivudine-induced mitochondrial myopathy. J Neurol Sci. 2015;358:481–3. 10.1016/j.jns.2015.08.1550 26359855

[23] Register SD, Aaron ME, Gelly HB. Hyperbaric oxygen therapy and optic neuritis: case report and literature review. Undersea Hyperb Med. 2011;38:557–9. 22292261

[24] Pell M, Saththasivam P, Stephens PL, Mychaskiw 2nd G. Therapeutic effect of hyperbaric oxygen on inclusion body myositis. Undersea Hyperb Med. 2012;39:1111–4. 23342768

